# Familial Identification: Population Structure and Relationship Distinguishability

**DOI:** 10.1371/journal.pgen.1002469

**Published:** 2012-02-09

**Authors:** Rori V. Rohlfs, Stephanie Malia Fullerton, Bruce S. Weir

**Affiliations:** 1 Department of Integrative Biology, University of California Berkeley, Berkeley, California, United States of America; 2 Department of Bioethics and Humanities, University of Washington, Seattle, Washington, United States of America; 3 Department of Biostatistics, University of Washington, Seattle, Washington, United States of America; Georgia Institute of Technology, United States of America

## Abstract

With the expansion of offender/arrestee DNA profile databases, genetic forensic identification has become commonplace in the United States criminal justice system. Implementation of familial searching has been proposed to extend forensic identification to family members of individuals with profiles in offender/arrestee DNA databases. In familial searching, a partial genetic profile match between a database entrant and a crime scene sample is used to implicate genetic relatives of the database entrant as potential sources of the crime scene sample. In addition to concerns regarding civil liberties, familial searching poses unanswered statistical questions. In this study, we define confidence intervals on estimated likelihood ratios for familial identification. Using these confidence intervals, we consider familial searching in a structured population. We show that relatives and unrelated individuals from population samples with lower gene diversity over the loci considered are less distinguishable. We also consider cases where the most appropriate population sample for individuals considered is unknown. We find that as a less appropriate population sample, and thus allele frequency distribution, is assumed, relatives and unrelated individuals become more difficult to distinguish. In addition, we show that relationship distinguishability increases with the number of markers considered, but decreases for more distant genetic familial relationships. All of these results indicate that caution is warranted in the application of familial searching in structured populations, such as in the United States.

## Author Summary

The forensic identification of criminal suspects through DNA profiling is now common in the United States. Indirect identification by familial DNA profiling is increasingly proposed to extend the utility of DNA databases. In familial searching, a DNA profile from a crime scene partially matches a database profile entry, implicating close relatives of the partial match. While the basic principles behind familial searching methods are simple and elegant, statistical confidence that a partially matched profile belongs to a true genetic relative has not been fully explored. Here, we derive relative identification likelihood ratio statistics and consider how the ability of familial searching to distinguish relatives from unrelated individuals varies over population samples and is affected by inaccurately assumed population background. We observe lower relationship distinguishability for population samples with less identifying information in the genetic loci considered. Additionally, we show that relationship distinguishability decreases with discordance between true and assumed population samples. These results indicate that, if an inappropriate genetic population group is assumed, individuals from certain marginalized groups may be disproportionately more often subject to false familial identification. Our results suggest that care is warranted in the use and interpretation of familial searching forensic techniques.

## Introduction

Forensic identification via exact genetic profile matching has become common practice in the United States [1]. In exact genetic identification, genetic markers found in a crime scene sample are genotyped and exactly matched to a suspect or database entry, suggesting that the sample originates from the matched individual. In some cases, a database search yields no exact genetic profile matches, but does reveal partial matches where some, but not all, alleles match. A partial match could result from a genetic familial relationship between the individual who left the sample and the database entrant. If the database entrant has relatives, they might be investigated to determine if any of their genetic profiles exactly match the sample.

Familial searching is now used fairly frequently in the United Kingdom and was instrumental in the identification of suspects of violent crimes for 20 cases lacking other evidence as of 2008 [2]. Its use in the United States has been more limited due to concerns regarding civil liberty infringement, racial bias, and efficacy [3]–[6]. However, in July 2010, familial searching was used in a highly publicized California case to identify a suspect serial killer (the “Grim Sleeper”) [7]–[10].

Despite the increasing use of familial searching in the United States, important questions about the method remain on both social and scientific grounds. In order to understand these concerns, we must appreciate that familial searching is most useful as a database mining method in cases with no suspects. In the United States, the Combined DNA Index System (CODIS) is the Federally administered system for National DNA Index System (NDIS), the national offender/arrestee database, which includes entries from State DNA Index Systems [11]. CODIS has standardized the use of genotypes at 13 particular short tandem repeats (STRs) (the CODIS loci) in forensic identification. The CODIS loci were chosen based on several criteria including reliable multiplexed PCR amplification, availability of commercial genotyping kits, clearly distinguishable alleles, linkage equilibrium, Hardy-Weinberg equilibrium, and high polymorphism in examined population samples [12]–[15]. An NDIS entry contains CODIS loci genotypes and a traceable index number, without other identifying information (e.g. location, race, or ethnicity) [16]. In September 2011, NDIS included over 10 million genotype profiles and continues to grow through new cases and expanded inclusion criteria [1].

These features of the forensic testing landscape matter because, unlike exact DNA identification, a typical database search for familial matches prospectively identifies candidate suspects who, while closesly genetically related to database entrants, are not in themselves in the database, provoking complex privacy concerns [4], [5], [9], [17], [18]. Additionally, social groups which both share genetic relationships and are over-represented in the database would experience a disproportionate increase in genetic surveillance if familial matching were routinely implemented, further exacerbating their over-representation in these databases [6], [12], [17]–[19].

The question of relative inference has been well-studied in other contexts with varying marker types, relationships, and numbers of individuals [20]–[28]. Here we focus on statistical and population genetic assumptions underpinning the familial searching methodology in the forensic context. Specifically, we consider the effects of both uncertainty in allele frequency estimation and population structure. First note that allele frequency estimates calculated within socially-defined population groups (e.g. African American, European American, Latino) are used to estimate the probability of an observed partial match, assuming a particular genetic relationship. Match probabilities for some individuals may not be accurately estimated using the available categorical socially-defined population group model and sample allele frequency data, particularly individuals with genetic ancestry outside of typically studied groups or individuals whose socially-defined population group does not inform their genetic ancestry. In exact identification, the probability of observing two individuals with identical specific 13-locus genotypes is astronomically low, with the exception of monozygotic twins. With these extremely low probabilities, differences or inaccuracies in allele frequency estimates are almost inconsequential, possibly changing the probability of an observed genotype a few orders of magnitude, but unlikely to alter the conclusion of the statistical analysis [29]. However, in familial identification, the probability of observing a coincidental partial match is much higher (e.g. for a parent-offspring relationship exactly one allele is shared by descent per locus). With these higher probabilities, population genetic differences in marker informativeness and errors in allele frequency estimation can perturb match probability estimations to such a degree as to affect the interpretation outcome.

In this study, we aim to examine some of these concerns by exploring how familial searching techniques behave on populations with varying allele frequency distributions and varying accuracy of allele frequency specification. We formulate and calculate confidence intervals for familial identification likelihood ratio (LR) estimates, and investigate how well siblings and unrelated individuals can be distinguished over different population samples with varying allele frequency distributions and under accurately and inaccurately assumed allele frequency distributions. We show that population samples vary in the amount of identifying information encoded in the CODIS loci and, therefore, in relationship distinguishability, even with correctly specified allele frequencies. Since completely accurate allele frequency specification is not guaranteed and the most appropriate population sample may not be known or available, we are also interested in the systematic effects of assuming allele frequencies which are appropriate for one population, but which are not appropriate for the individuals investigated. We show that relationship distinguishability decreases with the accuracy of allele frequency estimates, potentially resulting in high rates of coincidental familial identification for some groups. These results are especially pertinent in the multiple testing context of large database searching. In addition, we explore the relationships between relationship distinguishability, the number and type of markers used for identification, the relationship considered, and the true and assumed coancestry coefficient parameter value.

## Results

### Likelihood ratios for relationships with confidence intervals

To determine if a partial genotype match is better explained by a genetic familial relationship or stochasticity, we used the ratio of the likelihood of the observed partial match assuming the individuals share a given genetic familial relationship, to the likelihood of the observed partial match assuming the individuals are unrelated. With the data available, this LR is the most powerful statistic to separate relatives from unrelated individuals [30]. So even though the exact methodology used by forensic agencies for familial forensic identification is not readily publicly available, our use of the LR optimistically assumes the most powerful method using the CODIS loci. In the first part of this analysis, only sibling relationships are evaluated to reduce dimensionality. Other genetic familial relationships were explored and are reported below.

Unrelated individuals were simulated in a randomly mating population by independently drawing alleles from allele frequency distributions, similarly to Bieber *et al.* [31]. Siblings were then simulated by dropping alleles through a pedigree with unrelated parents. We simulated both unrelated individuals and siblings using allele frequency distributions from five socially-defined population samples, Vietnamese, African American, European American, Latino, and Navajo. Using both unrelated individuals and siblings, we calculated the sibling relationship 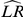 and 95% confidence interval of that estimate, assuming allele frequencies from each population sample. We simulated siblings and unrelated individuals under each of the five allele frequency distributions and calculated 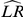 and 95% confidence interval of that estimate assuming each of the five allele frequency distributions 10,000 times for each pair of population samples. As a result, we have 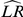 with confidence intervals for sibling relationships between unrelated individuals and siblings simulated from every population sample, assuming allele frequencies from every population sample. In most of the analyses presented here, we focus specifically on the lower 95% confidence limit of 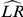 (LCL) to account for sampling and biological variance in allele frequency estimation and to conservatively identify relationships. We refer to the population sample used to simulate the individuals as the true population sample, as opposed to the assumed population sample used to calculate the LR for their relationship. Figure S1 shows the 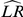 95% confidence intervals for 100 simulations of unrelated individuals, where individuals were simulated based on each population sample and confidence intervals were computed assuming the allele frequency distribution of each population sample.

Note that across all of these simulations specific parameter values were chosen and kept constant, specifically, sibling relationships, the assumed coancestry coefficient (probability of two alleles being identical by descent (IBD) between two individuals not recently related) used in calculations of 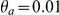, confidence interval length parameterized by significance level 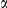 as 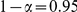, and the use of the 13 CODIS STRs. Regardless of the values of these parameters, the relative trends across true and assumed population samples will be maintained, although the scale may vary with parameter value choice.

#### Distinguishing relatives and unrelated individuals

**Figure 1:**
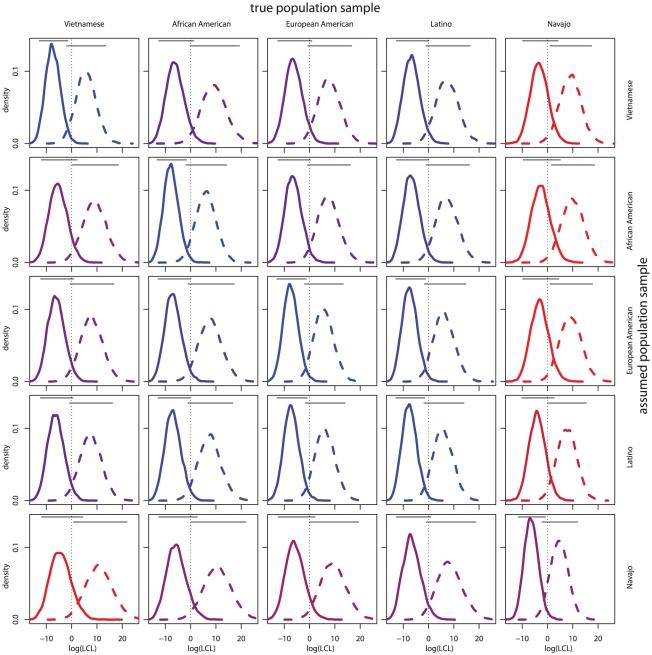
LCL distributions for siblings and unrelated individuals by population samples. Each individual plot shows the distribution of 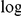 LCLs for unrelated individuals (solid) and siblings (dashed). The dotted vertical lines indicate 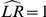. The horizontal lines show the central 95% of observed values over genotypes. The true and assumed population samples are listed on the column and row headings, respectively. Plot coloring indicates distinguishability where red represents low 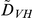 and blue represents high 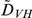.

To understand the degree to which 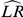 distinguishes relatives and unrelated individuals, we considered the distributions of LCLs for sibling relationships on simulated siblings and unrelated individuals. Figure 1 shows the density plots of the 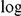 LCL for both siblings and unrelated individuals using different true and assumed population samples. First we consider plots along the diagonal of Figure 1 showing density curves for unrelated individuals and siblings when the true allele frequency distributions are assumed. Plots with more overlap between the sibling and unrelated pair densities indicate less ability to distinguish relatives from unrelated individuals, a feature we term distinguishability, for the assumed and true population samples. Overlap can be observed visually in both density curve overlap and the bars above the density curves which show the simulated empirical central 95% of LCL over genotypes. To quantify the differences in distinguishability between population sample pairs, 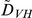 measures the distinctness of the distributions of LCLs for individuals who are truly unrelated and truly siblings (see Methods). Table 1 shows 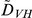 over true and assumed population samples. When the true population sample is assumed, 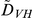 ranges from 5.87 for the Navajo sample to 7.38 for the African American sample (Table 1).

**Table 1:** 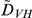
 between population samples.

	True population sample				
	Vietnamese	African American	European American	Latino	Navajo
Vietnamese	6.94	6.35	6.19	6.51	5.00
African American	5.91	7.38	6.41	6.59	4.68
European American	6.23	6.64	6.98	6.90	4.71
Latino	6.31	6.80	6.83	7.07	5.21
Navajo	5.01	5.94	5.74	5.94	5.87

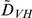
 between each true (columns) and assumed (rows) population sample pair calculated using the CODIS loci.

#### Gene diversity and distinguishability

Differences in distinguishability between population samples are rooted in differences in the shapes of allele frequency distributions. Since alleles and individuals are simulated independently, varying distinguishability over populations cannot be due to varying consanguinity and must be attributed to varying allele frequency distributions. In the examined population samples, the shape of allele frequency distributions can vary substantially. As a dramatic, but atypical, example, Figure S2 shows the different shapes of allele frequency distributions of D3S1358 for each population sample. Generally, the Navajo sample, and to a lesser extent the Vietnamese sample, allele frequency distributions have lower variance than that of the other samples, though not typically to the extreme extent seen at D3S1358.

Intuitively, it is clear that a monomorphic locus contains no identifying information, while a locus with a unique polymorphism for every individual contains complete identifying information. Along this spectrum, a locus with a low-variance allelic type distribution is less identifying than a locus with a high-variance allele frequency distribution.

This concept of varying identifying information can be quantified as observed gene diversity (or equivalently, average expected heterozygosity) [32]

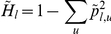
where 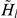 is the observed gene diversity for locus 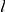 and 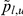 is the observed allele frequency of allele 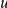 at locus 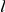. Observed gene diversity can be combined across loci as the mean of observed gene diversity at each individual locus to get average observed gene diversity 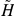. Using this method, we calculated the average observed gene diversity of the CODIS loci as 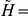
0.77, 0.79, 0.78, 0.79, and 0.70 for the Vietnamese, African American, European American, Latino, and Navajo samples, respectively (Text S1).

**Figure 2:**
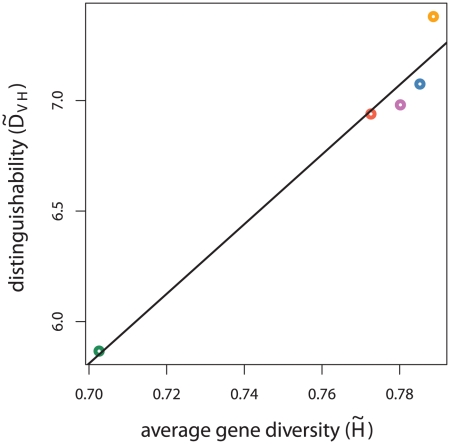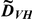 **versus** 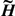 **.** The empirical distinguishability (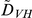) for siblings and unrelated individuals is plotted against average gene diversity (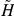) for each population sample. Points are colored according to the true population sample where red signifies Vietnamese, orange African American, purple European American, blue Latino, and green Navajo.

The calculated 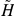 values show that the CODIS loci provide varying amounts of identifying information for different population samples. As our intuition suggests, population samples with lower-variance allele frequency distributions, particularly the Navajo sample, have lower average gene diversity. Even when assuming the correct allele frequency distribution, there is significant correlation between relationship distinguishability (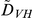) and average gene diversity (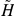) across population samples, as seen in Figure 2 (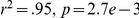).

Information theory can provide a more direct measure of identifying information through entropy, which we calculate to quantify the number of bits required to encode an equivalent amount of information as a CODIS haplotype for each population group. We find that relationship distinguishability is even more correlated with entropy than observed gene diversity, which follows since entropy quantifies information content which directly affects distinguishability (see Text S1 and Figure S3).

#### Allele frequency misspecification and distinguishability

By calculating LCL under different assumed and true population sample allele frequencies, the relationship between allele frequency misspecification and relationship distinguishability can be examined. By looking at plots and values off the diagonal, Figure 1 and Table 1, it is clear that distinguishability is particularly low when the true sample is Navajo and the assumed sample is different. This indicates that unrelated Navajo individuals more often appear sibling-like when non-Navajo allele frequencies are assumed. The same is true for the Vietnamese sample, though the trend is less pronounced.

**Figure 3:**
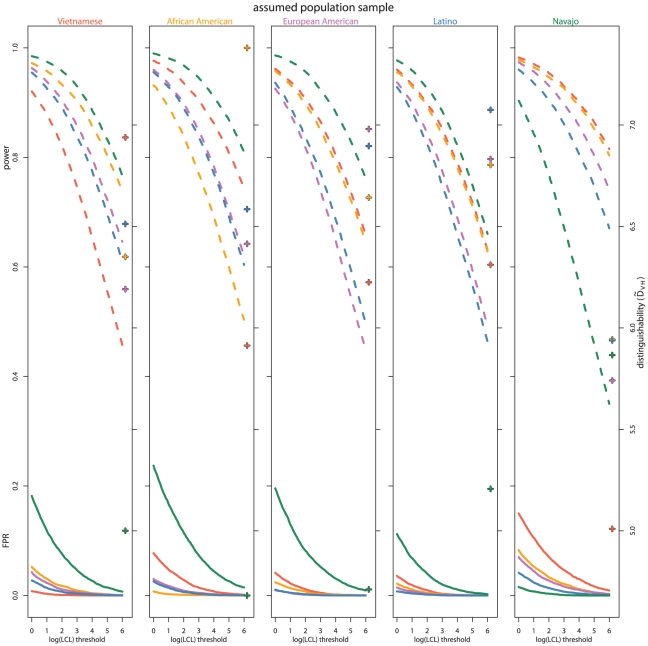
Power and false positive rate over thresholds and by population samples. The empirical power (dashed) and false positive rate (solid) are shown for a range of sibling versus unrelated 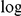 LCL decision thresholds. In each plot, the indicated population sample is assumed in the calculations. Within each plot, the colored curves indicate the true population sample allele frequencies used to simulate individuals. Red signifies Vietnamese, orange African American, purple European American, blue Latino, and green Navajo. Similarly color-coded crosses indicate 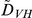 for each population sample pair.

In this study, we chose not to define a single decision threshold for determining positive relative identifications since such a threshold depends on a number of factors beyond the scope of this study (e.g., the social, economic, and political cost of false positives and negatives). For a range of decision thresholds, Figure 3 shows the false positive rate and the power. To intuitively calibrate 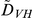 by commonly-used statistics, Figure 3 plots 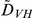 along with each set of false positive rate and power curves. False positive rate and power vary by population, with the true Navajo and assumed non-Navajo samples having particularly high false positive rates for decision thresholds shown. If a high decision threshold is chosen so that the false positive rate for true Navajo cases is comparably low as it is for other population samples, the power to identify siblings drops to levels that may render the investigation ineffective. In Figure 3 this can be visualized by choosing a point on the x-axis where the Navajo sample false positive rate is low (perhaps a decision threshold of 6) and looking up to the power to detect relationships at that threshold. A similar, but less pronounced, pattern appears with the Vietnamese data.

#### Low nominal false positive rates

It is notable that even when the correct allele frequencies are used, the false positive rate is lower than the 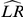 confidence interval significance level 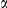. However, this is not surprising since the parameter 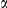 determines the width of the 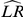 confidence interval, not the false positive rate. The confidence interval describes uncertainty in the LR estimation due to variance in the allele frequencies. In contrast, the false positive rate is a function of the low probability that two unrelated individuals share alleles in a pattern that resembles sibling relationships, which is often lower than the unrelated 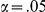 parameter value used here. See Text S1 for more details.

### 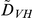 and 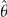


We observed lower distinguishability when the true and assumed allele frequency distributions differ more. The degree of difference between population sample allele frequency distributions at the CODIS alleles is quantified for every population pair using 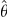 (Table 2). To account for multiple alleles at multiple loci and varying sample sizes, we estimate 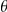 with the method of Weir and Cockerham [33]. Note that 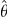
s reported here were calculated using the only CODIS loci, as is appropriate for an analysis of forensic methods. For a thorough investigation of the population genetics of these samples, more loci would be required, producing different results than those shown here, as reported in other studies [34], [35].

**Table 2:** 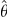
 between population samples.

	African American	European American	Latino	Navajo
Vietnamese	0.038	0.026	0.021	0.068
African American		0.017	0.015	0.067
European American			0.000	0.059
Latino				0.041

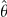
 between each population sample as calculated using the CODIS loci. Estimates less than 0.0 are reported as 0.0.

**Figure 4:**
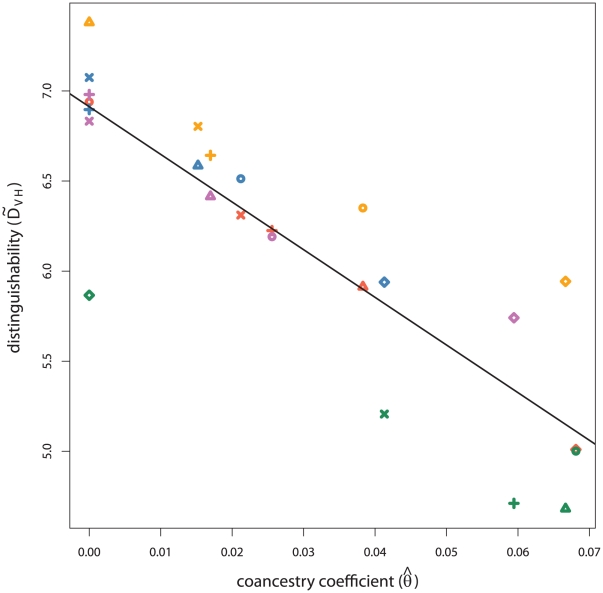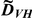 **versus** 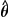 **.** The empirical measure of distinguishability (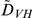) for siblings and unrelated individuals is plotted against 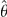 for each pair of true and assumed population samples. Points are colored according to the true population sample and take a shape according to the assumed population group where red and circles signify Vietnamese, orange and triangles African American, purple and plus marks European American, blue and multiplication marks Latino, and green and diamonds Navajo. 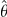 estimates less than 0.0 are reported as 0.0.

To explore the relationship between distinguishability and the genetic distance between true and assumed population samples, in Figure 4, 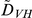 is plotted against 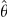 for each pair of true and assumed population samples. 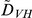 and 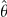 are significantly correlated (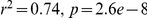), supporting the hypothesis that incorrectly assuming allele frequencies leads to low distinguishability and high false positive rates. In particular, we observe low distinguishability when Navajo, or to a lesser extent Vietnamese, is the true population sample, correlating with higher 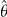 with the other assumed samples.

Intuitively, when allele frequencies are misspecified, the most likely error is assuming that common alleles are more rare simply because truly common alleles are more likely to be observed than truly rare alleles. In the same way, rare alleles are assumed to be common, but by definition, rare alleles are less likely to be observed shared between individuals, so overall the misspecification of common alleles as rare dominates. When misspecifying common alleles as rare, observing the same common alleles in multiple individuals seems surprising, so a genetic relationship model is favored over a model of no relationship. That is, the probability of a partial match assuming a relationship is inflated and the probability of a partial match assuming no relationship is deflated. In this way, allele frequency misspecification results in an increase in false positive relative identifications.

Although the relationship between distinguishability and allele frequency misspecification has not yet been deeply considered in the context of genetic familial identification (but see [36]), it has been discussed in the forensic literature for exact matching and it is well-known in the linkage analysis community. For exact forensic identification using the 13 CODIS loci, discrepancies between assumed and true allele frequencies affect the computed match probabilities, but seldom change the ultimate outcome [37]–[40]. In linkage analysis, when inaccurate population allele frequencies are used to calculate genotype probabilities, false linkage signals between genotype and phenotype are common [41], [42].

#### Additional populations

We have shown clear differences in average observed gene diversity of the CODIS loci and resulting differences in sibling and unrelated individual distinguishability in the five population samples considered. To ensure that these findings extend beyond the samples examined, we considered a larger dataset with a total of 32 population samples [43]. As in the five-population sample dataset, average observed gene diversity at the CODIS loci varies between samples, with particularly low values for Native American samples (Text S1). We performed a comparable analysis of average observed gene diversity versus distinguishability using ten population samples and found again that 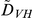 is correlated with 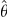 over true and assumed population samples (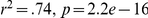, Figure S4).

### Distinguishability over parameters

In the analysis presented thus far, we showed how distinguishability varies over true and assumed population samples with varying allele frequency distributions. To maintain manageable dimensionality, some key parameters likely to vary in forensic analyses were kept constant. Here we explore the relationships between these parameters, particularly different genetic relationships, varying marker data, and varying the true and assumed coancestry coefficients (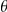 and 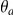). To focus on the relationships between these parameters, in these analyses the correct known allele frequencies were used.

Pairs of individuals were simulated taking into account the true coancestry coefficient, 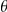, using the genotype probabilities described in the Text S1, for the following genetic relationships: parent-offspring, sibling, half-sibling, first cousin, second cousin, and unrelated. Note that in contrast with the analyses presented above, here 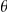 is used to model background relatedness. LRs were computed comparing the probabilities of the simulated data assuming the true relationship and assuming the individuals are unrelated. This analysis was repeated over varying numbers and types markers and a variety of assumed 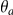 values.

#### Varying number and type of markers

We simulated two types of markers with equi-frequent alleles: 10-allele STRs and 2-allele SNPs. We varied the number of simulated markers over 10, 20, 30, 40, 50, and 60 STRs and 10, 50, 100, 150, 200, and 250 SNPs in independent simulations. Distinguishability between the LCL distributions of true relatives and unrelated individuals were calculated for each of these simulations (Figure S5). Distinguishability varies widely over relationships, with sibling 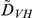 being two or three orders of magnitude higher than second cousin 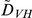. We also see distinguishability increase with the number of markers.

For unrelated individuals, 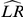 for a parent-offspring relationship is often exactly 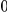 since unrelated individuals are unlikely to share at least one allele at each locus. As a result, the distribution of 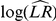 is not definable and distinguishability cannot be computed, so parent-offspring relationships are excluded from these results.

#### Varying 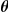 and 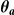


The genetic similarlity of relatives can be quantified with the kinship coefficient, which is the probability that a pair of alleles from relatives are IBD. The kinship coefficient for parent-offspring, sibling, half-sibling, first cousin, and second cousin relationships are 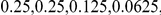 and 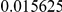, respectively. Intuitively, as the kinship coefficient of the tested relationship approaches the population background relatedness (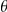), it will become increasingly difficult to discern relatives from unrelated individuals.

To explore the relationship between true coancestry coefficient 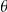, assumed coancestry coefficient 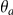 used in probability calculations, genetic similarity of relatives, and 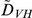, we consider 15 STRs and 100 SNPs and simulated individuals with true population 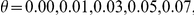 and 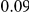. We then calculated LRs using 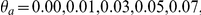 and 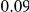. For each type of marker, distinguishability decreased as 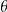 increased and the slope of that decrease flattens as 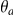 increased (Figure S6). Again, distinguishability varied over relationships where 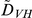 for siblings was about three orders of magnitude greater than 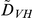 for second cousins. This consistent with findings by Anderson and Weir that IBD sharing estimation accuracy increased with the number of markers considered and decreased as 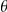 increased [44].

## Discussion

The analysis presented here confirms and quantifies the intuition from population genetics that for particular loci, groups with comparatively low-variance allele frequency distributions have less identifying information encoded in genotypes. Decreased identifying information results in lower relationship distinguishability, even when the correct allele frequency estimates are used (Figure 2, Figure S2). This is abundantly apparent for the Native American samples considered in this analysis.

With a basic understanding of population genetics, it is clear that socially defined groups, like Navajo, Latino, or European American, have very different underlying population structures reflecting distinct demographic history, degrees of genetic diversity, and admixture. It is hardly surprising that a group which has undergone multiple population size reductions, like the Navajo, has a lower-variance allele frequency distribution than a group with a history of genetic diversity and social inclusion, like African Americans. This is particularly evident at the CODIS loci, which were chosen in part because of their broad allele frequency distributions in a few studied populations, without considering all relevant populations [13]–[15].

These population differences in allele frequency distributions are key when considering a potential source of error: inappropriately assumed allele frequency distributions. When the allele frequency distributions for an inaccurately specified population group are assumed, the probabilities of the observed data under a sibling relationship and under no close genetic relationship become less distinct, so relationship distinguishability decreases. We found that distinguishability decreases with increased distance between assumed and true allele frequency distributions, as measured through 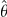. Specifically, both Navajo and Vietnamese samples are more genetically distant to the other three samples considered and show decreased distinguishability when allele frequencies of one of those three samples are assumed.

The results of this analysis indicate that when a decision threshold is chosen so that the power to identify siblings is reasonably high, population samples with allele frequencies which differ from those assumed would experience disproportionately higher rates of false positive familial identification (Figure 3). This could be exacerbated by unknown population-based differences in genotyping which would distort allele frequencies, for example, population-specific mutations in PCR primer binding sites [45]–[51]. More extensive genotyping of genetically diverse populations may make available more appropriate allele frequency distributions. However, it is not clear how or if the most appropriate allele frequency distribution for a pair of samples can be determined. Population-based differential distinguishability will persist, regardless of additional population-specific allele frequency distributions or uniformly applied corrections. One possible correction would be increasing the value of the parameter 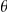, however, in Figure S6 we see that even when the true allele frequencies are assumed, increasing 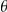 decreases distinguishability. If more genetic data were used, particularly markers on the Y chromosome or mitochondrial DNA, as are in some states but not Federally, profile informativeness could be increased to the point where allele frequency approximations made little difference in the ultimate outcome (Figure S5) [10], [52]. However, additional Y chromosome and mitochondrial markers will only inform matrilinial or patrilinial relationships and any additional markers will be subject to similar population-specific variation, and will be limited by practical genotyping constraints and the need to avoid medically-associated regions. Additionally, it is not clear if more distant relationships (cousins, second cousins, etc) would be confidently identified, even with more independent genetic loci (Figure S5) [53], [54]. As it is, the core 13 CODIS loci, or the minimum 10 loci recommended by the Scientific Working Group on DNA Analysis Methods Ad Hoc Committee on Partial Matches (SWGDAM), seem inadequate to implement sibling matching with low false positive rate and high power in structured populations [52], [55]. More complex situations, like mixed or low-template DNA samples, require further study and may not be feasible with the 13 CODIS loci [55], [56].

Motivated by the question of forensic familial searching, in this analysis we focus on distinguishing relatives with a specified relationship and unrelated individuals. In other contexts, it may be more appropriate to distinguish different kinds of relatives (e.g. siblings and parent-offspring) or relatives with an unspecified relationship and unrelated individuals. In the former case, the ratio of LRs for the relationships of interest versus unrelated individuals reduces to the LR comparing the two specified relationships. In the later case, models allowing IBD sharing probabilities to vary can be formulated and incorporated into the LR. For example, when comparing a null model with set IBD sharing probabilities for unrelated individuals and an alternative where the likelihood of data is maximized over any IBD sharing probabilities, a LR test can be formulated which follows a 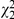 distribution under the null hypothesis.

This analysis considers familial identification in a forensic context, but is applicable to tests for relatedness applied in the various contexts especially when considering unlinked genetic markers as in paternity investigation, ecological surveys, and conservation biology. When more extensive genotype or sequence data are available, it is appropriate to use more sophisticated tests for relatedness considering linkage or shared haplotype length [28], [57], [58].

The population genetic model used in forensic identification is remarkably coarse. In direct identification, the CODIS loci provide ample data to determine identity and non-identity, even with the coarse population genetic model of a small number of discrete homogenous genetic groups corresponding to social racial groups. We have shown that under this model, new concerns arise with familial searching. However, the model itself requires some scrutiny. It is clear that human genetic population structure is complex and humans are not easily split into a small number of discrete homogenous genetic groups [59]–[62]. Even with carefully chosen and defined population samples, it is practically impossible to account for human genetic variation and the discrete population group model fails to account for individuals with mixed ancestry. Additionally, individuals are typically assigned to genetic population groups based on social race. While there is correlation between genetic ancestry and social race, one does not determine the other [63]. As a result, in the discrete population group model, some individuals may not be grouped with the most similar genetic group.

Forensic familial searching will most likely be implemented in the context of a large offender/arrestee database, introducing questions of multiple testing over both database entrants, and the number of genetic familial relationships considered. Because forensic methodology practice varies over jurisdictions, it is not clear how these multiple testing issues have been, or will be, addressed. However, it is reasonable to assume that familial searching will result in a list of partial database matches with 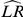 for genetic familial relationships. The parameter values used in the 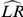 calculations must be conservative to keep the number of high 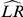 partial matches manageably short, but the parameters also must allow enough leniency so that a true match will appear in the list considered. Ideally, parameter values used in practice should be tuned using simulations based on real genotype data representing realistic cryptic relatedness and population structure appropriate to the database and relevant population. When tuning parameters, as power increases, false positive rate will as well. Both of these values must be considered in deciding on appropriate parameter values. However across parameter values, some groups may have higher rates of false identification, as we have shown here, raising questions about the practicality of familial searching. Without access to accurate database or population information, or to a clear decision procedure practice, we refrain from making specific recommendations about parameter choice or methodology in this analysis.

Individual and population genotype information is necessary to determine the extent to which inaccurately assumed allele frequencies cause high false positive rate in familial matching in practice. For instance, in this study, we considered unrelated individuals, conforming to exactly one of five allele frequency distributions, in completely randomly mating populations. However the use of familial searching rests on the premise that relative groups are in the database and population structure is undeniably present in most databases [64]. Access to suitably secure and encrypted database information would enable analyses with an accurate portrayal of relatedness and population substructure. As recommended by Krane *et al.*, increased transparency in database makeup, search procedure, and database access are required for rigorous analyses of forensic methodology [65].

If implemented with the core CODIS loci, familial searching may result in low distinguishability and potentially high false positive rates among certain groups, especially if only African American, European American, Southeastern Latino, and Southwestern Latino allele frequency distributions are in assumed LR calculations, as recommended by SWGDAM [55]. Because some of these groups (Native Americans and some immigrant groups) are correlated with social groups already over-represented in the criminal justice system, group members would be more likely to have a relative in the database, and that relative would be more likely to have a coincidental partial match with a crime scene sample [3]–[6], [9], [17], [18], [66]–[68]. Cumulatively, members of these groups are more likely to be investigated as a familial match due to over-represention in the database, and an unusually high false positive familial identification rate.

## Methods

### Data

Our analysis makes use of allele frequency data for the 13 CODIS loci over different population samples socially defined by race. Note that alternate schemes to group individuals will also produce genetic differences between groups [56], [63], [69]. Here, we consider genetic differences between socially-determined groups which are relevant to the practice of genetic familial forensic identification. To do so, we used the allele frequencies reported by Budowle and Moretti [29] for samples from ‘Vietnamese,’ ‘African American,’ ‘Caucasian,’ ‘Hispanic,’ and ‘Navajo’ populations. In this manuscript, these same samples are refered to with the following labels: Vietnamese, African American, European American, Latino, and Navajo. As short hand, we refer samples derived from individuals from each sample as the sample name, for example ‘the Latino sample.’ The number of individuals genotyped to estimate allele frequencies for each sample varied, with 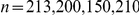, and 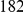 individuals sampled for Vietnamese, African American, European American, Latino, and Navajo samples, respectively.

The consent and population grouping procedures used in obtaining these data are not clear. In the time since these data were collected, dominant cultural ethics regarding informed consent process have changed considerably, motivated largely by several cases of severe misuse of samples provided by Indigenous communities [70]–[73]. As a result, today it is becoming less acceptable to gather data in the same way [74]–[78]. We use the data because of its public availability, however we look forward to working with data collected using transparent informed consent methodology.

### Likelihood ratio for relationship

LRs are used to compare the probability of observed genotypes for two individuals under two different hypotheses: the individuals are unrelated (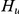) and the individuals share a specified genetic familial relationship (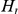) [79]. The LR is defined as [79]

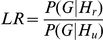
where 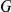 is the observed pair of genotypes. When 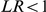, the observed data are more likely for unrelated individuals and when 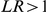, the observed data are more likely for individuals with the specified genetic relationship.

By assuming independence between all CODIS loci, 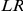 can be broken down as
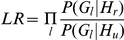
where 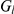 is the observed genotype for each individual at locus 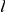.

Relationships between individuals can be described using the identical by descent (IBD) sharing probabilities 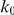, 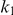, and 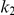, which are the probabilities that individuals with the specified relationship share 0, 1, and 2 alleles IBD, respectively [79]. For example, for a parent/offspring relationship 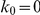, 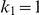, and 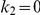 and for a sibling relationship 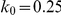, 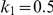, and 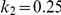.

Using these IBD sharing probabilities, the LR becomes
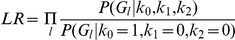
where the IBD sharing probabilities in the numerator are specified by the specific genetic relationship considered. The probability of the observed genotype combinations given IBD sharing probabilities depends on the specific combination of alleles observed. The probabilities of all observed genotypes, given IBD sharing probabilities, are defined in Text S1. These probabilities include a correction for expected background relatedness using the coancestry coefficient 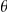. In the first part of this study, we use the value of 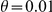 based on standard methodology in population genetics and as recommended by SWGDAM [55], [80].

### Likelihood ratio confidence intervals

The LR described above provides information about whether the observed data are more likely for unrelated or related individuals. However, the true population allele frequencies (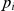) are unknown, so 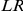 needs to be estimated with the observed allele frequencies. Available sample allele frequencies are subject to sampling variation and variation due to demographic history [81]. Observed allele frequencies follow directly from observed genotype frequencies. Using 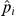, the probability of the data is calculated under different IBD sharing schemes, so the estimate of the likelihood ratio (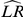) can be computed. By considering the distribution of 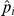, we can find the distribution of 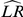 and calculate confidence intervals on reported 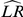 values.

Sampling variation is inherent in allele frequency estimation since a random sample must be chosen for the estimate. By their nature, different random samples vary in their representation of specific alleles, resulting in different allele frequency estimates. Additionally, random genetic sampling exists in the historical differentiation of populations, resulting in population groups with distinct allele frequencies. Since all present-day human population groups descend from a common ancestral population, the alleles present in each present-day population group reflect a sample of the alleles from the common ancestral population.

Under evolutionary equilibrium and a simple model of demographic history, the relationship between population group allele frequencies (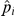) can be modeled using a Dirichlet distribution informed by the coancestry coefficient (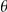), accounting for genetic and sampling variation in estimated allele frequencies [81], [82]. With this model, we define the 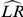 confidence interval in order to express uncertainty conferred by allele frequency estimate.

Using the same approach as Beecham and Weir [81], we note that the total 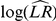 is the sum of the 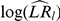 for each locus 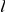. The central limit theorem indicates that, for even as few as 13 independent loci, this sum will be approximately normally distributed [81]. Thus, the confidence interval for 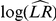 is [81]

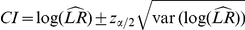
where 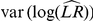 is the variance of 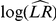 and 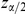 is the standard normal value for the given 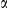, in this study 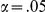 and so 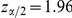. While the typical arbitrary value of 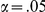 is used in this study, the trends explored will be maintained with different values of 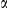. Also note that a one-sided confidence interval can be derrived similarly with 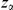. This confidence interval is in 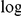 space, so we can exponentiate the results to get the confidence interval of 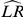. The value of 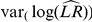 (derived in Text S1) depends on the variances of the observed allele frequencies. These, in turn, depend on 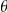 to accommodate evolutionary variation over populations and this is why numerical techniques such as bootstrapping cannot be used to calculate likelihood ratios, as explained by Beecham and Weir [81].

### Simulating individuals

Using the data provided by Budowle and Moretti [29], individuals were simulated based on the allele frequencies reported for each of the five population samples. For the population structure analysis, individuals are simulated from a given population sample by independently drawing two alleles from the appropriate allele frequency distribution for every locus. Note that the total independence between drawn alleles implicitly creates a population with a coancestry coefficient of zero (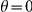). Independently generated individuals are unrelated. Related individuals are simulated by generating unrelated individuals and randomly dropping alleles through a pedigree to achieve the desired relationship. In this way, we simulate pairs of both unrelated and related individuals from each population sample.

The total lack of population structure or cryptic relatedness (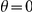) in our simulated populations causes unrelated individuals to share fewer alleles than would be expected in a real population. This contrasts with our use of the 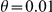 correction in 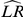 calculations, conservatively lowering our calculated 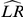. This is consistent with forensic applications, where a conservatively high value for 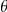 is chosen for the anticipated populations. Specifically, 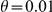 and 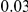 have been suggested for use with populations primarily of European and Native American descent, respectively [43], [83].

In the second part of this analysis, when we consider the interplay between various parameters, it is necessary to simulate unrelated individuals from a population with a given non-zero coancestry coefficient (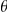). To simulate unrelated and related individuals from a population with 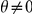, random alleles are drawn using the probabilities of two-individual genotypes, given 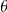 and a specified relationship, as written in Text S1.

### Comparative distribution analysis

We are interested in comparing LCL distributions generated with different parameters, particularly LCL distributions for truly unrelated individuals and truly related individuals. If the relationship 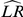 perfectly distinguished relatives and unrelated individuals, these two distributions would be totally separate. The degree of overlap between the related and unrelated distributions roughly indicates the degree of genetic similarity of relatives and unrelated individuals, and so, how well 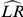 distinguishes the two.

To quantify distinguishability, we use an empirical version of the measure proposed by Visscher and Hill [56]

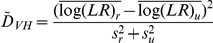
where 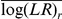 and 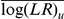 are the sample means of 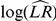 for the simulations of related and unrelated individuals, respectively, and 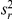 and 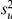 are the sample variances of 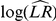 for the simulations of related and unrelated individuals, respectively. Note that 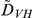 is analogous to the non-centrality parameter of the LR test statistic distribution under the alternative hypothesis. Higher 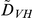 indicates greater LR distribution differentiation and more distinguishability, while lower 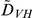 indicates more overlap and less distinguishability. The statistic 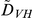 accurately describes the differentiation in LR distributions, and is particularly appealing because it describes the difference in distributions, so it does not rely on a parameterized decision procedure to discretely determine relationship status.

## Supporting Information

Figure S1Confidence intervals by population samples. Each plot shows the 100 replicates of 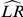 95% confidence intervals for a sibling relationship between unrelated individuals, assuming allele frequencies based on the named population sample. Within each plot, the colored bands show the population sample allele frequencies used to simulate the unrelated individuals. Red signifies Vietnamese, orange African American, purple European American blue Latino, and green Navajo. The vertical line indicates 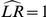.(EPS)

Figure S2 Allele frequency distributions. Each plot shows the D3S1358 allele frequency distribution for each population.
(EPS)

Figure S3

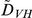
 versus entropy. The empirical distinguishability (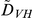) is plotted against entropy for each population sample.(EPS)

Figure S4Distinguishability (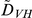) versus distance between true and assumed population samples (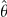). The empirical distinguishability (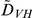) is plotted against 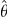 for each pair of true and assumed population samples. Points are colored according to the true population sample in the stated color scheme. 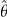 estimates less than 0.0 are reported as 0.0.(EPS)

Figure S5

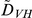
 over number of markers and relationships. 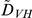 is shown when simulating different numbers of STRs (first column) and SNPs (second column) for a variety of relationships, as labeled.(EPS)

Figure S6

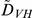
 over 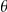, 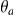, and relationships. 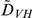 is shown when simulating 15 STRs (first column) and 100 SNPs (second column) with different values of 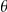 used in the simulation and 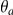 used in probability calculations for a variety of relationships, as labeled.(EPS)

Text S1Supporting work is presented, specifically genotype probability equations, 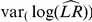 derivation, low nominal false positive rates, relationship distinguishability and entropy, and tables.(PDF)
