# Supplementary material for: Familial Identification: Population Structure and Relationship Distinguishability
Source: PLoS Genet. 2012 Feb 9;8(2):e1002469. doi: 10.1371/journal.pgen.1002469 (PMC3276546; doi:10.1371/journal.pgen.1002469)
Supplement: Text S1 — Supporting work is presented, specifically genotype probability equations, derivation, low nominal false positive rates, relationship distinguishability and entropy, and tables. (PDF) [file pgen.1002469.s007.pdf]

Supporting information for
Familial identification: Population structure and relationship distinguishability

Genotype probabilities

The probabilities of different observed genotypes for two individuals, given a set of kinship coefficients are as follows [1]

$$\begin{aligned}
P(A_1A_1, A_1A_1|k_2, k_1, k_0) &= k_2(p_1(\theta + (1-\theta)p_1)) \\
&+ k_1(p_1(\theta + (1-\theta)p_1)(\frac{2\theta + (1-\theta)p_1}{1+\theta})) \\
&+ k_0(p_1(\theta + (1-\theta)p_1)(\frac{2\theta + (1-\theta)p_1}{1+\theta})(\frac{3\theta + (1-\theta)p_1}{1+2\theta})) \\
P(A_1A_1, A_1A_2|k_2, k_1, k_0) &= k_1\frac{p_1p_2(1-\theta)(\theta + (1-\theta)p_1)}{\theta+1} \\
&+ k_0\frac{2p_1p_2(1-\theta)(\theta + (1-\theta)p_1)(2\theta + (1-\theta)p_1)}{(\theta+1)(2\theta+1)} \\
P(A_1A_2, A_1A_2|k_2, k_1, k_0) &= k_2 2p_1p_2(1-\theta) \\
&+ k_1\frac{p_1p_2(1-\theta)(2\theta + (1-\theta)(p_1+p_2))}{\theta+1} \\
&+ k_0\frac{4p_1p_2(1-\theta)(\theta + (1-\theta)p_1)(\theta + (1-\theta)p_2)}{(\theta+1)(2\theta+1)} \\
P(A_1A_2, A_1A_3|k_2, k_1, k_0) &= k_1\frac{p_1p_2p_3(1-\theta)^2}{\theta+1} \\
&+ k_0\frac{4p_1p_2p_3(1-\theta)^2(\theta + (1-\theta)p_1)}{(\theta+1)(2\theta+1)} \\
P(A_1A_1, A_2A_2|k_2, k_1, k_0) &= k_0\frac{p_1p_2(1-\theta)(\theta + (1-\theta)p_1)(\theta + (1-\theta)p_2)}{(\theta+1)(2\theta+1)} \\
P(A_1A_1, A_2A_3|k_2, k_1, k_0) &= k_0\frac{2p_1p_2p_3(1-\theta)^2(\theta + (1-\theta)p_1)}{(\theta+1)(2\theta+1)} \\
P(A_1A_2, A_3A_4|k_2, k_1, k_0) &= k_0\frac{p_1p_2p_3p_4(1-\theta)^3}{(\theta+1)(2\theta+1)}
\end{aligned}$$

where p_i is the allele frequency for observed allele A_i and θ is the coancestry coefficient used to account for population structure.

Note that in these calculations, allele frequencies between populations are assumed to follow a Dirichlet distribution. Under this model, the probability of observing the allele A_i depends on the number previous observations n_i as

$$P(A_i|n_i) = \frac{n_i\theta + (1-\theta)p_i}{1 + (n-1)\theta}$$

where n is the total number of alleles observed and p_i is the allele frequency. The use of the coancestry coefficient θ accounts for variation in allele frequencies among population samples.

Computing $\mathrm{var}(\log(\widehat{LR}))$

We apply the methodology of Beecham and Weir [2] to compute the variance of the $\log(\widehat{LR})$, by first considering that if the alleles at each locus are independent,

$$\mathrm{var}(\log(\widehat{LR})) = \sum_{l} \mathrm{var}(\log(\widehat{LR}_l))$$

The $\mathrm{var}(\log(\widehat{LR}_l))$ term can be approximated using the Taylor expansion so that

$$\begin{aligned}\mathrm{var}(\log(\widehat{LR}_l)) &= \sum_{i=1}^{m} \left(\left(\frac{\partial \log(\widehat{LR}_l)}{\partial \hat{p}_i} \right)^2 \mathrm{var}(\hat{p}_i) \right) \\ &\quad + \sum_{i=1}^{m} \left(\sum_{j=2, j \neq i}^{m} \left(\frac{\partial \log(\widehat{LR}_l)}{\partial \hat{p}_i} \frac{\partial \log(\widehat{LR}_l)}{\partial \hat{p}_j} \mathrm{cov}(\hat{p}_i, \hat{p}_j) \right) \right)\end{aligned}$$

where m is the number of observed alleles at locus l and $\hat{p}_i$ and $\hat{p}_j$ are the estimated allele frequency at locus l for alleles i and j. The variance and covariance of allele frequency estimations are obtained using the underlying Dirichlet model as [3]

$$\begin{aligned}\mathrm{var}(\hat{p}_i) &= \hat{p}_i(1 - \hat{p}_i)((2n - 1)\theta + 1)/(2n) \\ \mathrm{cov}(\hat{p}_i, \hat{p}_j) &= -\hat{p}_i\hat{p}_j((2n - 1)\theta + 1)/(2n)\end{aligned}$$

where n is the number of individual genotypes used to calculate $\hat{p}_i$ and $\hat{p}_j$ and θ is the coancestry coefficient. The partial derivative of $\log(\widehat{LR})$ can be written as

$$\begin{aligned}\frac{\partial \log(\widehat{LR}_l)}{\partial \hat{p}_i} &= \frac{\partial(\log(P(G_l|H_r)) - \log(P(G_l|H_u)))}{\partial \hat{p}_i} \\ &= \frac{1}{P(G_l|H_r)} \frac{\partial \log(P(G_l|H_r))}{\partial \hat{p}_i} \frac{1}{P(G_l|H_u)} \frac{\partial \log(P(G_l|H_u))}{\partial \hat{p}_i}\end{aligned}$$

Partial derivatives

The partial derivatives of the probability the observed genotype, assuming a set of kinship coefficients are as follows.

$$
\begin{aligned}
\frac{\partial P(A_1A_1, A_1A_1|k_2,k_1,k_0)}{\partial p_1} &= k_2(\theta + 2p_1(1-\theta)) \\
&+ k_1(p_1(\theta + (1-\theta)p_1)\frac{1-\theta}{1+\theta} + \frac{2\theta + (1-\theta)p_1}{1+\theta}(\theta + 2p_1(1-\theta))) \\
&+ k_0(p_1(\theta + (1-\theta)p_1)\frac{(2\theta + (1-\theta)p_1)(1-\theta)}{(1+\theta)(1+2\theta)} \\
&\quad +(p_1(\theta + (1-\theta)p_1)\frac{1-\theta}{1+\theta} + \frac{2\theta + (1-\theta)p_1}{1+\theta}(\theta + 2p_1(1-\theta)))\frac{3\theta + (1-\theta)p_1}{1+2\theta}) \\
\frac{\partial P(A_1A_1, A_1A_2|k_2,k_1,k_0)}{\partial p_1} &= k_1\frac{p_2(1-\theta)(\theta + 2p_1(1-\theta))}{\theta+1} \\
&+ k_0\frac{2p_2(1-\theta)(2\theta^2 + 6\theta p_1(1-\theta) + 3p_1^2(1-\theta)^2)}{(\theta+1)(2\theta+1)} \\
\frac{\partial P(A_1A_1, A_1A_2|k_2,k_1,k_0)}{\partial p_2} &= k_1\frac{p_1(1-\theta)(\theta + (1-\theta)p_1)}{\theta+1} \\
&+ k_0\frac{2p_1(1-\theta)(\theta + (1-\theta)p_1)(2\theta + (1-\theta)p_1)}{(\theta+1)(2\theta+1)} \\
\frac{\partial P(A_1A_2, A_1A_2|k_2,k_1,k_0)}{\partial p_1} &= k_2 2p_2(1-\theta) + k_1\frac{p_2(1-\theta)(2\theta + (1-\theta)(2p_1+p_2))}{\theta+1} \\
&+ k_0\frac{4p_2(1-\theta)(\theta + (1-\theta)p_2)(\theta + 2p_1(1-\theta))}{(\theta+1)(2\theta+1)} \\
\frac{\partial P(A_1A_2, A_1A_2|k_2,k_1,k_0)}{\partial p_2} &= k_2 2p_1(1-\theta) + k_1\frac{p_1(1-\theta)(2\theta + (1-\theta)(2p_2+p_1))}{\theta+1} \\
&+ k_0\frac{4p_1(1-\theta)(\theta + (1-\theta)p_1)(\theta + 2p_2(1-\theta))}{(\theta+1)(2\theta+1)}
\end{aligned}
$$

$$\frac{\partial P(A_1A_2, A_1A_3|k_2,k_1,k_0)}{\partial p_1} = k_1\frac{p_2p_3(1-\theta)^2}{\theta+1} + k_0\frac{4p_2p_3(1-\theta)^2(\theta+2p_1(1-\theta))}{(\theta+1)(2\theta+1)}$$

$$\frac{\partial P(A_1A_2, A_1A_3|k_2,k_1,k_0)}{\partial p_2} = k_1\frac{p_1p_3(1-\theta)^2}{\theta+1} + k_0\frac{4p_1p_3(1-\theta)^2(\theta+(1-\theta)p_1)}{(\theta+1)(2\theta+1)}$$

$$\frac{\partial P(A_1A_2, A_1A_3|k_2,k_1,k_0)}{\partial p_3} = k_1\frac{p_1p_2(1-\theta)^2}{\theta+1} + k_0\frac{4p_1p_2(1-\theta)^2(\theta+(1-\theta)p_1)}{(\theta+1)(2\theta+1)}$$

$$\frac{\partial P(A_1A_1, A_2A_2|k_2,k_1,k_0)}{\partial p_1} = k_0\frac{p_2(1-\theta)(\theta+(1-\theta)p_2)(\theta+2p_1(1-\theta))}{(\theta+1)(2\theta+1)}$$

$$\frac{\partial P(A_1A_1, A_2A_2|k_2,k_1,k_0)}{\partial p_2} = k_0\frac{p_1(1-\theta)(\theta+(1-\theta)p_1)(\theta+2p_2(1-\theta))}{(\theta+1)(2\theta+1)}$$

$$\frac{\partial P(A_1A_1, A_2A_3|k_2,k_1,k_0)}{\partial p_1} = k_0\frac{2p_2p_3(1-\theta)^2(\theta+2p_1(1-\theta))}{(\theta+1)(2\theta+1)}$$

$$\frac{\partial P(A_1A_1, A_2A_3|k_2,k_1,k_0)}{\partial p_2} = k_0\frac{2p_1p_3(1-\theta)^2(\theta+(1-\theta)p_1)}{(\theta+1)(2\theta+1)}$$

$$\frac{\partial P(A_1A_1, A_2A_3|k_2,k_1,k_0)}{\partial p_3} = k_0\frac{2p_1p_2(1-\theta)^2(\theta+(1-\theta)p_1)}{(\theta+1)(2\theta+1)}$$

$$\frac{\partial P(A_1A_2, A_3A_4|k_2,k_1,k_0)}{\partial p_1} = k_0\frac{p_2p_3p_4(1-\theta)^3}{(\theta+1)(2\theta+1)}$$

$$\frac{\partial P(A_1A_2, A_3A_4|k_2,k_1,k_0)}{\partial p_2} = k_0\frac{p_1p_3p_4(1-\theta)^3}{(\theta+1)(2\theta+1)}$$

$$\frac{\partial P(A_1A_2, A_3A_4|k_2,k_1,k_0)}{\partial p_3} = k_0\frac{p_1p_2p_4(1-\theta)^3}{(\theta+1)(2\theta+1)}$$

$$\frac{\partial P(A_1A_2, A_3A_4|k_2,k_1,k_0)}{\partial p_4} = k_0\frac{p_1p_2p_3(1-\theta)^3}{(\theta+1)(2\theta+1)}$$

Low nominal false positive rates

The false positive rate can be no higher than the rate at which unrelated individuals randomly share enough alleles to resemble the stated relationship, which is determined by the degree of matching required and the estimated allele frequencies. As a simple example, consider a parent-offspring relationship where individuals must share at least one allele at every locus. The probability of this degree of allele sharing between unrelated individuals is orders of magnitude lower than $\alpha = .05$. We computed the probability of two unrelated individuals sharing at least one allele at every locus, which would resemble a parent and offspring relationship, as shown below. These probabilities are 5.9e-8, 2.8e-8, 4.5e-8, 3.3e-8, and 7.9e-7 for each population sample in the order previously stated (Table S1).

The general familial identification false positive rate could be determined numerically by the expressions given in [4] for the probabilities that two individuals share zero, one or two pairs of alleles at a locus, whether or not they are related. Using these equations, evaluated with appropriate database allele frequencies, and parameters could be adjusted to attain a desired false positive rate and the expected power could be computed. In a database with unrelated individuals from multiple discrete population groups without population substructure, the overall false positive rate will be the sum of the product of

false positive rate and number of individuals present in the database for each population group. That is,

$$FPR_{total} = \sum_i FPR_i d_i$$

where FPR_i and d_i are the false positive rate and proportion of individuals in the database, respectively, for each population group i. However, in an actual database, the assumptions of discrete known population groups without cryptic relatedness or substructure will almost certainly not hold, increasing the actual false positive rate.

Frequency of parent-offspring-like genotypes

As shown in [4], the probability of two unrelated individuals sharing one allele at every locus, resembling a parent and offspring relationship is

$$\begin{aligned}
&\prod_l \sum_{u,a,b} P((A_{l,u}A_{l,a})(A_{l,u}A_{l,b})|k_0 = 1, k_1 = 0, k_2 = 0) \\
&= \prod_l \sum_u \Bigg(\sum_{a \neq u} \sum_{b \neq u,a} \frac{p_{l,u}p_{l,a}p_{l,b}(1-\theta)^2(\theta + (1-\theta)p_{l,u})}{(1+\theta)(1+2\theta)} \\
&\quad + \sum_{a \neq u} \frac{4p_{l,u}p_{l,a}(1-\theta)(\theta + (1-\theta)p_{l,u})(\theta + (1-\theta)p_{l,a})}{(1+\theta)(1+2\theta)} \\
&\quad + \sum_{a \neq u} \frac{p_{l,u}p_{l,a}(1-\theta)(\theta + (1-\theta)p_{l,u})(2\theta + (1-\theta)p_{l,u})}{(1+\theta)(1+2\theta)} \\
&\quad + \frac{p_{l,u}(\theta + (1-\theta)p_{l,u})(2\theta + (1-\theta)p_{l,u})(3\theta + (1-\theta)p_{l,u})}{(1+\theta)(1+2\theta)} \Bigg)
\end{aligned}$$

where $A_{l,u}$, $A_{l,a}$, and $A_{l,b}$ are alleles at locus l.

Relationship distinguishability and entropy

The amount of information which can be contained in random variables from given probability distributions can be quantified as entropy. In the context of genotypes, we write the entropy of locus l as

$$-\sum_u p_{l,u} log_2(p_{l,u})$$

where $p_{l,u}$ is the allele frequency of allele u at locus l. Conveniently, this entropy is the number of bits required to encode the information conveyed in locus l. Entropy can be combined across independent loci by simply summing the entropy at each individual locus. Using this method, we calculated the entropy of the CODIS loci for each population group as 31.8, 33.3, 32.0, 32.5, and 27.7 for the Vietnamese, African American, European American, Latino, and Navajo samples, respectively (Table S1). These entropy calculations show that the CODIS loci provide varying amounts of identifying information for different population groups. As our intuition suggests, population samples with low-variance allele frequency distributions have lower entropy.

Since entropy quantifies the information contained in a genotype which is used to distinguish relatives, we find that entropy and distinguishability are very well correlated ($p = 1.2e-4, r^2 = 0.99$), as illustrated in Figure S3.

References

[1] Lewis K (2009) Genomic Approaches to Forensic DNA Analysis. Ph.D. thesis, University of Washington.

[2] Beecham G, Weir B (2011) Confidence interval of the likelihood ratio associated with mixed stain DNA evidence. Journal of Forensic Sciences 56: S166-S171.

[3] Curran J, Triggs C, Buckleton J, Weir B (1999) Interpreting DNA mixtures in structured populations. Journal of Forensic Sciences 44: 987-995.

[4] Weir B (2007) The rarity of DNA profiles. The Annals of Applied Statistics 1: 358-370.

Supporting tables

	Median confidence interval length	Mean $\log(\widehat{LR})^2$	Probability of two unrelated individuals sharing at least one allele at every locus	Mean gene diversity	Entropy
Vietnamese	2.4e-3	52.2	5.9e-8	0.77	31.8
African American	1.8e-3	56.6	2.6e-8	0.79	33.3
European American	2.7e-3	52.2	4.5e-8	0.78	32.0
Latino	2.2e-3	54.2	3.3e-8	0.79	32.5
Navajo	6.1e-3	40.0	7.9e-7	0.70	27.7

Table S 1: Population sample statistics

Descriptive statistics for each of the examined population samples.

Population sample	Subpopulation sample	Average observed gene diversity ($\tilde{H}$)
African-descent	FBI	0.79
	Bahama	0.79
	Jamaica	0.78
	Trinidad	0.80
	California	0.79
	Alabama	0.79
	Florida	0.79
	Virginia	0.79
	New York	0.79
	Illinois	0.79
Asian-descent	Chinese	0.77
	Japanese 1	0.78
	Japanese 2	0.77
	Korean	0.77
	Vietnamese	0.77
	General Asian	0.79
European-descent	FBI	0.78
	California	0.79
	Alabama	0.78
	Florida	0.78
	Virginia	0.78
	Michigan	0.78
Latino-descent	FBI	0.77
	California	0.78
	Florida	0.79
	New York	0.80
	Michigan	0.79
	Arizona	0.79
	Mexico	0.78
Native American	Michigan	0.79
	American Indian tribe 1	0.71
	American Indian tribe 2	0.70

Table S 2: CODIS loci average observed gene diversity by population sample
